# A novel approach to the assessment of higher-order rule learning in male mice

**DOI:** 10.1101/lm.053771.123

**Published:** 2023-10

**Authors:** Renee Y. Chasse, Peter A. Perrino, Ruth M. McLeod, Gerry T.M. Altmann, R. Holly Fitch

**Affiliations:** 1 Department of Psychological Sciences, University of Connecticut, Storrs, Connecticut 06269, USA; 2 Murine Behavioral Neurogenetics Facility, Institute of Brain and Behavioral Sciences, University of Connecticut, Storrs, Connecticut 06269, USA

## Abstract

Historically, the development of valid and reliable methods for assessing higher-order cognitive abilities (e.g., rule learning and transfer) has been difficult in rodent models. To date, limited evidence supports the existence of higher cognitive abilities such as rule generation and complex decision-making in mice, rats, and rabbits. To this end, we sought to develop a task that would require mice to learn and transfer a rule. We trained mice to visually discriminate a series of images (image set, six total) of increasing complexity following three stages: (1) learn a visual target, (2) learn a rule (ignore any new images around the target), and finally (3) apply this rule in abstract form to a comparable but new image set. To evaluate learning for each stage, we measured (1) days (and performance by day) to discriminate the original target at criterion, (2) days (and performance by day) to get back to criterion when images in the set were altered by the introduction of distractors (rule learning), and (3) overall days (and performance by day) to criterion when experienced versus naïve cohorts of mice were tested on the same image set (rule transfer). Twenty-seven wild-type male C57 mice were tested using Bussey–Saksida touchscreen operant conditioning boxes (Lafayette Instruments). Two comparable black–white image sets were delivered sequentially (counterbalanced for order) to two identical cohorts of mice. Results showed that all mice were able to effectively learn their initial target image and could recall it >80 d later. We also found that mice were able to quickly learn and apply a “rule” : Ignore new distractors and continue to identify their visual target embedded in more complex images. The presence of rule learning was supported because performance criterion thresholds were regained much faster than initial learning when distractors were introduced. On the other hand, mice appeared unable to transfer this rule to a new set of stimuli. This is supported because visual discrimination curves for a new image set were no better than an initial (naïve) learning by a matched cohort of mice. Overall results have important implications for phenotyping research and particularly for the modeling of complex disorders in mice.

Traditionally, “ learning” is defined as the ability to acquire memories rapidly and efficiently through a learning environment (Saar and Barkai 2003). Most rodent models of higher-order cognitive learning have been developed using rats (Saar and Barkai 2003; Gilboa et al. 2014). This includes seminal studies of category learning, reversal learning, set shifting, and rule learning. Category learning—crucial to the maintenance of attention to category-relevant information and the updating of category representations—has been shown to depend on prelimbic circuits (Hamilton and Brigman 2015; Broschard et al. 2021). Reversal learning (the ability of rodents to switch preference to previously unrewarded strategies or stimuli) has been related to activation in the orbitofrontal cortex, dorsal striatum, and amygdala (Bissonette and Powell 2012; Hamilton and Brigman 2015). Set shifting (required when shifting attentional sets to new modalities or stimuli) has been shown to depend on medial wall structures such as the anterior cingulate, the prelimbic and infralimbic cortices, the amygdala, and the dorsomedial striatum (Bissonette and Powell 2012). Some rodent models have shown further evidence that higher-order learning depends on nondeclarative systems, such as motivational salience and reward value (Gilboa et al. 2014). Rule learning (the ability to learn and apply/transfer abstract rules to concrete stimuli) has been associated with activation in prefrontal regions (Peyrache et al. 2009), and it has been hypothesized that functional communication between the prefrontal cortex and the hippocampus is necessary for memory maintenance during rule-based learning (Peyrache et al. 2009). This link could also reflect dopamine signaling through reward-related pathways (Peyrache et al. 2009), consistent with evidence of correlations between the magnitude of increase of prefrontal dopamine and the learning rate in a rule-shifting task (Stefani and Moghaddam 2006). Such evidence suggests that rule learning might depend heavily on reward delivery and saliency relative to cognitive “strategy” in rodents (Broschard et al. 2019).

Despite knowledge gained from the rat studies described above, few of these higher-order tasks have been successfully translated to murine models (Graybeal et al. 2014; Yang et al. 2020). One barrier has been verifying the inferred use of specific cognitive processes, usually by eliminating possible alternate strategies. Although rats have shown marginal evidence of being able to learn and transfer information from one set to another (e.g., attentional set shifting), mice have shown limited abilities in this domain. However, studies of engineered mouse models for genetic disorders characterized by cognitive and language disruption would benefit greatly from the addition of such tasks to standard phenotyping batteries. Effective cross-species transfer would benefit efforts to phenotype transgenic murine models, a popular method for identifying genetic bases of disorders characterized by cognitive and developmental deficits. Enhanced assessment of such higher-order skills in mice could boost the clinical significance of this basic research. In the current study, we used a novel touch screen-based paradigm in which mice were required to discriminate a visual stimulus set using a reward/time-out scheme and then apply this rule to a novel stimulus set. If the mice were able to learn the “rule” and apply it to brand new stimuli, then this would suggest that evaluation of such higher-order cognition in mice is possible, supporting future use as a new phenotyping measure.

## Results

Performance on target discrimination in preswitch stage A showed that all subjects were able to learn their target by reaching a criterion level of >75% correct for two consecutive days, consistent with ample prior evidence that mice are able to discriminate two distinct and standard visual images from each other following protocols used here (Horner et al. 2013; Truong et al. 2014; Rendall et al. 2019; Chasse et al. 2021). Moreover, no differences were detected between performances as a function of the target on the final day of preswitch stage A (*F*_(1,26)_ = 0.198, *P* = 0.660), signifying comparability of target difficulty. These results show that subjects were able to satisfy step 1 of the task. Moreover, by the end of the final day of the overall task (preswitch stage C), all mice were scoring >75% correct responses, again regardless of image set. This shows that criteria used during each phase of shaping, training, and testing successfully advanced subjects to the most difficult stages of the task given that premature advancement would lead to failed performance on more difficult stages.

When comparing performance across stages A–C within image sets, we consistently saw that subjects reached criterion faster by orders of magnitude in stages B and C compared with A. Groups took from 15 to 23 d to reach 75% on stage A under both preswitch and postswitch conditions (23 d for circle/cross and 15 d for heart/star). Conversely, groups took only a few days to reattain 75% levels when stimuli were shifted to stages B and C (all sets). This shows that mice did not treat images containing both target and distractor(s) as “new” complex images, which would trigger new learning and expected learning curves comparable with those in stage A. Rather, days to criterion were more than halved in stage B, showing instead that subjects were able to learn a simple “rule” to ignore any distractor image(s) and focus on the target alone. Thus, mice were able to satisfy step 2 of the task.

**Figure 1.**
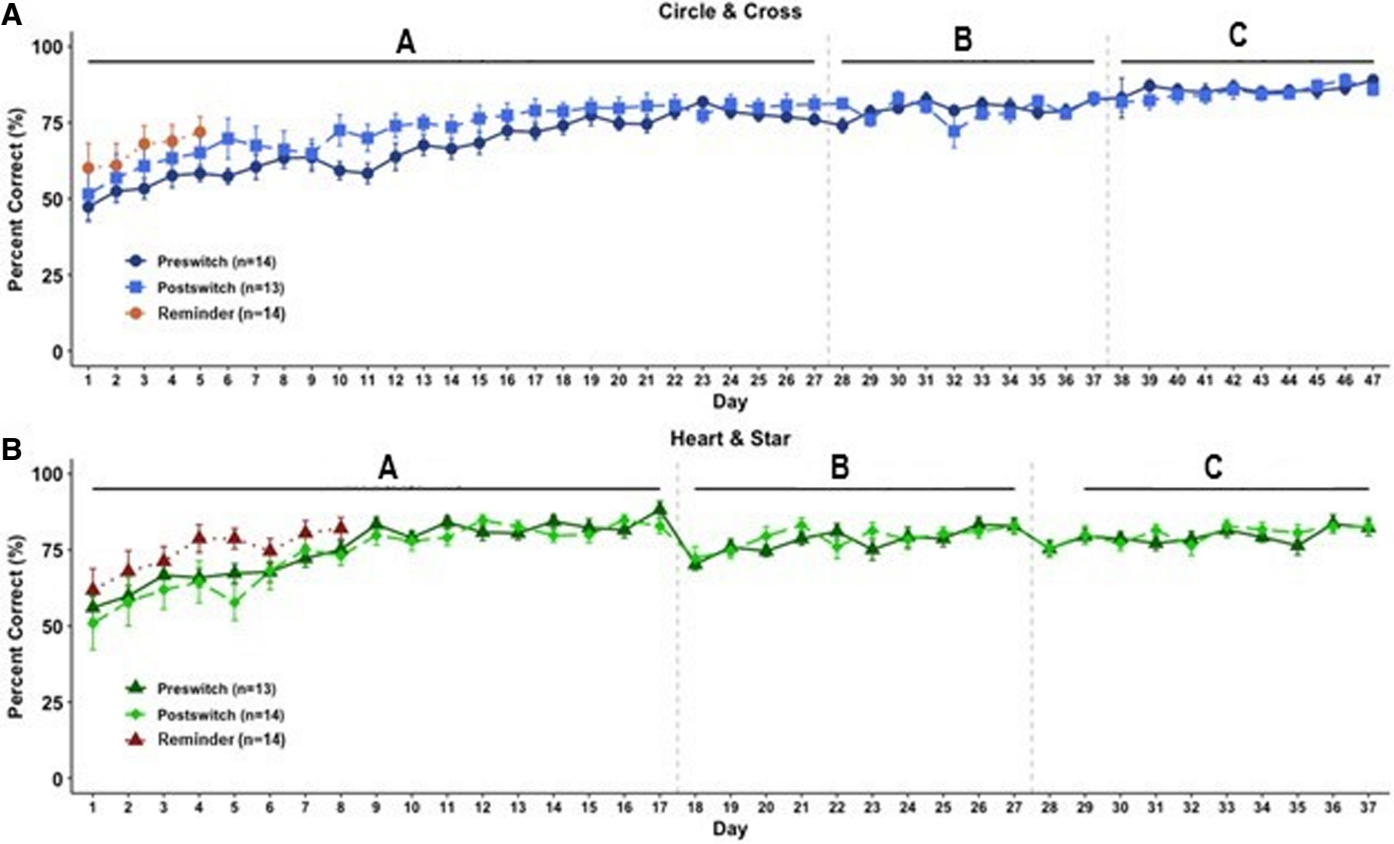
Pairwise discrimination performance across stages as grouped by stimuli presented and order of presentation. Overall percent correct was analyzed by day. (*A*) Circle/cross; *n* = 14 for preswitch and reminder groups; *n* = 13 for postswitch group. (*B*) Heart/star; *n* = 13 for preswitch and reminder groups, *n* = 14 for postswitch group. Data shown are mean ± SEM for each group.

**Figure 2.**
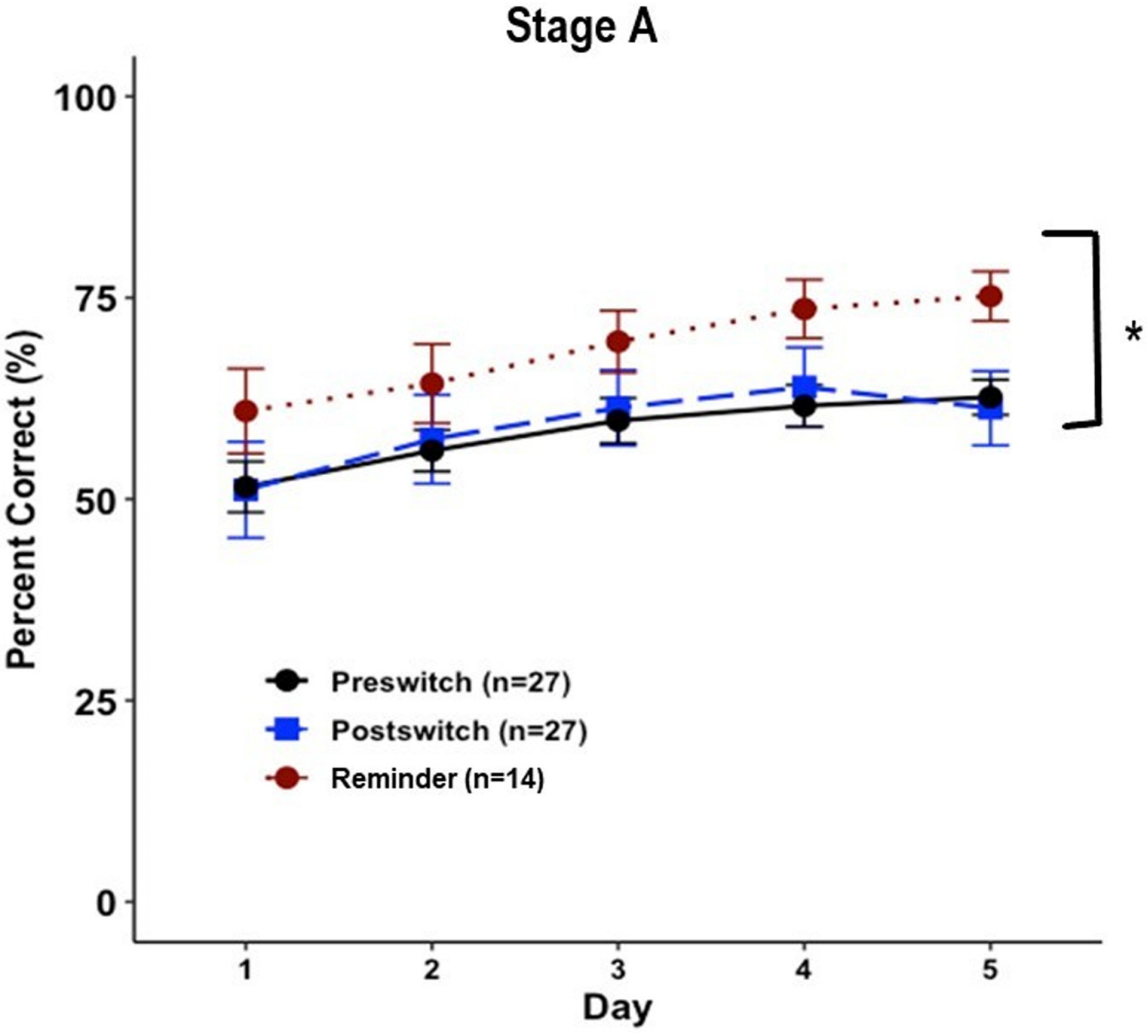
Pairwise discrimination performance across stages as grouped by stimuli presented and order of presentation. Overall percent correct was analyzed by day. (*) *P* < 0.05. Data shown are mean ± SEM for each group.

**Figure 3.**
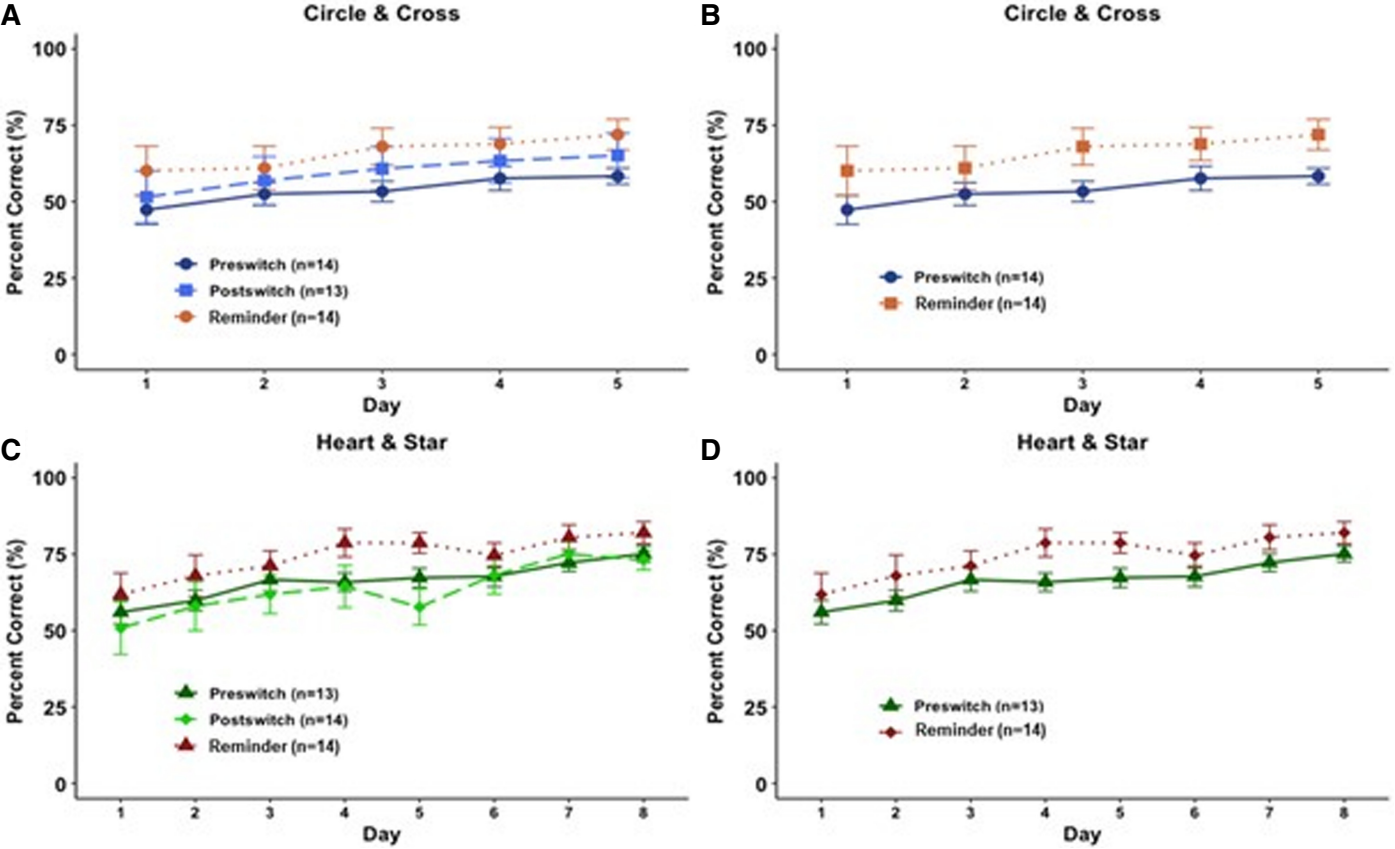
Pairwise discrimination performance across stages as grouped by stimuli presented and order of presentation. Overall percent correct was analyzed by day. (*A*) Circle/cross; *n* = 14 for preswitch and reminder groups; *n* = 13 for postswitch group. (*B*) Circle/cross; *n* = 14. (*C*) Heart/star; *n* = 13 for preswitch and reminder groups; *n* = 14 for postswitch group. (*D*) Heart/star; *n* = 13. Data shown are mean ± SEM for each group.

When comparing preswitch and postswitch performance across both image sets, a repeated measures ANOVA showed no significant differences in overall performance based on stimulus order presentation (preswitch vs. postswitch). This result indicates a lack of any benefit from prior exposure to the task (circle/cross, preswitch vs. postswitch: *F*_(1,25)_ = 1.13, *P* = 0.30; heart/star, preswitch vs. postswitch: *F*_(1,25)_ = 0.03, *P* = 0.87) (Fig. 1A,B). A similar lack of effects was seen when comparing within individual stages; stage A showed no significant difference between preswitch (naïve learning) and postswitch (experienced; circle/cross: *F*_(1,25)_ = 1.92, *P* = 0.18; heart/star: *F*_(1,25)_ = 0.33, *P* = 0.57) (see Figs. 1–3). Likewise, we saw no experience effects on stage B (circle/cross: *F*_(1,25)_ = 0.09, *P* = 0.77; heart/star: *F*_(1,25)_ = 0.14, *P* = 0.71) (Fig. 1A,B) or stage C (circle/cross: *F*_(1,25)_ = 0.22, *P* = 0.64; heart/star: *F*_(1,25)_ = 0.11, *P* = 0.74) (Fig. 1A,B). Another way to assess learning is to compare days to criterion (75% correct). During postswitch learning, groups took the same number of days to reach the criteria, with no evidence of benefit from prior experience on the other image set. Overall results show that postswitch performance was identical to that of mice who were naïve, showing that adult C57 mice could not satisfy step 3 of the task.

Interestingly, the reminder stage (which returned to the original image set) revealed a modest benefit from prior stimulus exposure even though the original preswitch stimulus exposure (either heart/star or circle/cross) had occurred almost 80 d prior (circle/cross: *F*_(1,26)_ = 3.17, *P* = 0.09; heart/star: *F*_(1,24)_ = 2.99, *P* = 0.10) (Figs. 1A,B, 2, 3A–D). This benefit of prior exposure on performance was significant when the image set groups were combined (*F*_(1,52)_ = 5.38, *P* < 0.05) (Fig. 2).

## Discussion

The purpose of this study was to address the limited availability of assessments to index higher-order cognition in mice. Such tasks would have tremendous value for basic research on animal models for disorders characterized by subtle cognitive and/or language impairments. A secondary purpose was to validate two new sets of visual stimuli in a pairwise discrimination touchscreen model. Although our study showed that C57 mice do not appear to be capable of abstract rule transfer (i.e., the ability to use prior experience to inform a future discrimination with novel images), we did validate that more complex visual discrimination sets can be used to show rule learning in mice. Such tasks can offer a more sensitive litmus for phenotyping higher-order disorder animal models as compared with a simple single-object discrimination.

Results from our initial stage of pairwise discrimination learning (preswitch A) confirmed historic research showing that mice can discriminate between two standardized and visually equivalent but distinct images to identify and learn a target. Moving on to preswitch B, subjects were required to distinguish their target image from a distractor image (another image included with their target). Subjects were successfully able to ignore the distractor as well as additional distractors introduced in preswitch C. Subjects showed robust performance with each new stage of distractors, regaining criterion in fewer than half the days required to learn the target in stage A. This reflects an ability to learn a simple rule: Ignore visual distractors (Fig. 1A,B).

Once the preswitch stages were complete, subjects were required to learn an entirely new correct target image in postswitch A (Fig. 1A,B). Here, we tested the hypothesis that subjects could take the rule learned with the prior target and transfer it, presumably in abstract form, to an entirely new image set. If true, subjects should move through subsequent stages in fewer days than naïve animals learning that same image set (17 d for heart/star and 27 d for circle/cross). Our results did not bear this out, however. Instead, the C57 mice required just as many days to discriminate the new target and reach criteria on subsequent stages as the naïve animals, indicating that they treated the new task as entirely new and not just a new image set in the same task (Fig. 1A,B). Performance in postswitch B and C was equivalent to that of preswitch B and C, confirming a lack of abstract rule transfer.

Overall, our findings are in accord with evidence from previous studies (Peyrache et al. 2009; Broschard et al. 2019). Our results show that mice can learn what appears to be a “concrete” rule (i.e., a rule applicable to specific stimuli; ignore distractors and focus on a learned target). They did not, however, appear to be able to transfer an abstract version of that rule to a new image set. Nonetheless, use of preswitch stages A–C alone could be highly applicable to studies of loss of function in brain regions known to mediate attention and behavioral flexibility, such as the prefrontal cortex and locus coeruleus. Despite our failure to demonstrate abstract rule transfer, the preswitch task alone provides a way to tap the ability to learn a simple rule that requires filtering information and focusing on a target embedded among distractors. Such a task could provide insights into the workings of genetic risk factors for developmental disorders that include atypical attention or cognitive processing such as autism spectrum disorder, dyslexia, or attention deficit/hyperactivity disorder.

One interesting result of this study is that the distinction between the heart/star image sets was “easier” for C57 mice to learn than the circle/cross image sets. The reason for this is unclear, since stimuli were equiluminant, but it could reflect subtle spatial high-frequency differences between the shapes or species-specific predilections for certain visual patterns. Nonetheless, our design was balanced to account for this variation by comparing preperformance and postperformance on the same image sets. Despite differences in days to criterion between sets, identical rule learning and a lack of rule transfer results were seen within image sets.

Another interesting result from this experiment was that mice were able to remember their initial target stimuli after almost 80 d of testing on another set of images (Figs. 1A,B, 2). This evidence of long-term memory could inform future studies; for example, by using this paradigm as a tool to measure long-term memory retention in models of Alzheimer's or other memory disorders.

Limitations of this experiment include that mice sharing a C57 background do have a history of blindness in older age, so the likelihood of completing a visual task in extremely aged animals may be limited. Nonetheless, they performed in the current paradigm without evidence of visual decline up to 129 d of age. According to prior studies, this strain of mice shows declines in visual discrimination ability between 12 and 17 mo of age (Buscher et al. 2017); these mice were significantly younger, therefore mitigating the concern of age-related visual acuity decline. Additionally, the possibility of overtraining is a concern with such extended behavioral testing. To address this issue, we used a more modest learning criteria (75%) in the testing stages and required subjects to sustain this performance over 2 d (thus capturing evidence of ceiling performance without overtraining).

Additionally, the choice to single-house testing subjects is one that could introduce confounding data into the study. Many studies (Gordon et al. 1998; Kercmar et al. 2011; Liu et al. 2020; Capoferri et al. 2021; Park et al. 2021) have shown cognitive impairments and depressive-like symptoms in single-housed animals, which may limit the scope of these results. Additionally, data have shown that the c57BL/6J strain has decreased novel object recognition when single-housed versus group-housed (Voikar et al. 2005), although it is important to note that visual pairwise discrimination measured through the use of operant conditioning and novel object recognition use different learning circuits in the brain (Lee et al. 2021; Hiramoto et al. 2023; Ottenheimer et al. 2023). However, contradicting data have shown that group-housed animals did not perform as many trials per day as single-housed animals (Reinert et al. 2019), and that is a metric of particular importance in this task. Ultimately, there is no substantial evidence detailing the differences between single and group housing on operant conditioning, which points to another potential study focus for future researchers.

A follow-up study to this one could investigate the ability of mice to perform incongruent switches, where instead of the correct image staying the same, the incorrect image remains but the correct image (target) changes. Based on results from work with mice learning reversals and stimulus switches, such incongruent switches may be difficult for mice to perform; if even possible, it would take subjects an extended period of time. Nonetheless, this would be an interesting study to determine whether mice are attending to stimuli considered “incorrect” rather than testing attention to and identification of target (correct) stimuli.

In summary, the current study aimed to develop a higher-order task in mice that might tap abstract rule learning and transfer using a visual pairwise discrimination task in touch screen operant conditioning boxes. The study found evidence of visual rule learning but not abstract rule transfer in mice. The results provide a platform for further task development in mice to expand the battery of tasks applicable to phenotyping models of cognitive and language disability.

## Materials and Methods

All experimental procedures in this study were performed in compliance with National Institutes of Health regulations and were approved by the Institutional Animal Care and Use Committee of the University of Connecticut.

### Subjects

Wild-type male c57BL/6J mice (*n* = 28) were obtained through the Jackson Laboratory (000664; https://www.jax.org/strain/000664). During all behavioral testing, subjects were single-housed in standard mouse tubs under a 12-h/12-h light/dark cycle. This was necessary to regulate the amount of food individually consumed during restriction. However, mice were provided with small huts and cotton nesting material as enrichment. Fourteen days before the start of operant conditioning training (P37), mice began food restriction. Although this age is younger than usual for food restriction, the lengthy testing demands coupled with visual loss in C57 mice starting at ∼6 mo required us to begin as early as possible. Body weights were gradually reduced to 85% baseline, as adjusted for age based on the vendor's standard weight curves. Since all mice returned to full weight under ad libitum feed at the end of the study, we can confirm this protocol did not lead to early growth restriction. Seven days prior to training, subjects were given a sample (∼1 mL) of the liquid food reward (Strawberry Ensure Plus, Abbott Nutrition) in their home cage. This continued for five continuous days. Pairwise discrimination testing began at P65. All behavioral testing occurred during the light phase.

Subjects were assessed for visual discrimination in automated Bussey–Saksida touch screen operant conditioning boxes (Lafayette Instruments). Each box had a trapezoidal operant area, a touch screen (30.7 cm, resolution 800 × 600), and a liquid-dispensing unit situated across from the center of the screen. On the touch screen, a cover prevented mice from touching the screen anywhere except for two panels situated on two separate halves of the screen. For all tasks conducted in the operant conditioning boxes, a flavored nutritional liquid food reward (Strawberry Ensure Plus, Abbott Nutrition) was provided at 20 µL per correct response.

### Pretraining (P44)

Each subject had one training session (60 min or until a maximum of 30 rewards were dispensed signaling 30 correct answers) each day for 10 d.

#### Must initiate

During the first 5 d of pretraining, mice learned to initiate the presentation of the stimulus on the screen by a nose poke in the reward delivery tray, exiting the reward tray (as recorded by a break in an infrared beam), and then touching a stimulus presented on the screen to obtain a food reward. At this stage, touches to areas of the screen with no stimulus present were not registered. Criterion was established when mice completed 30 trials.

#### Punish incorrect

During the next 5 d of pretraining, mice learned to touch only the stimulus presented on the screen and not a blank area of the screen. In this phase, the concept of an incorrect answer and a punishment was added. On the screen, one side of the screen had a stimulus presented and one side did not, instead showing a blank portion of the screen. Touching this blank screen was considered an “incorrect” response and was punished by a time-out for 5 sec, during which no inputs were registered and the operant area was brightly illuminated by a house light (∼60 lux). Following the time-out period, the reward tray light would turn on and the animal was allowed to begin the next trial. This phase captured the number of correct responses, the number of incorrect responses, and the total duration of each session. Criterion was established when mice reached 80% correct responses.

### Pairwise discrimination (P65)

Once an animal initiated a trial (as described in “Must Initiate”), two novel white stimuli (shapes) were presented on either side of the black screen. These preconstructed stimuli were the product of extensive testing by the manufacturer to be visually equivalent (luminance, pixels, etc.) yet distinct. Animals were pseudorandomized to determine which image was assigned as target. If the animal selected the correct image, a food reward was presented in the reward tray. If the animal selected the incorrect image, there was a time-out period of 5 sec during which no touches on the screen or nose pokes into the reward tray were registered, as well as a flash of the house light (as described in “Punish Incorrect”). All subjects repeated this sequence for a total of 30 trials per session, completing one session per day. This task recorded the total duration of each session, as well as the percent of correct responses made by the animal. Once animals reached the criterion of at least 70% of correct responses, they were moved to the rule-learning task. Subjects who were unable to reach criterion (*n* = 1) were removed from the study.

### Rule-learning task

Here we trained mice to visually discriminate a series of images (image set, six total) of increasing complexity following three stages: (1) learn a visual target (preswitch stage A), (2) learn a rule (ignore any new images around the target; preswitch stages B and C), and finally (3) apply this rule in abstract form to a comparable but new image set (postswitch stages A–C).

#### Group determination

**Figure 4.**
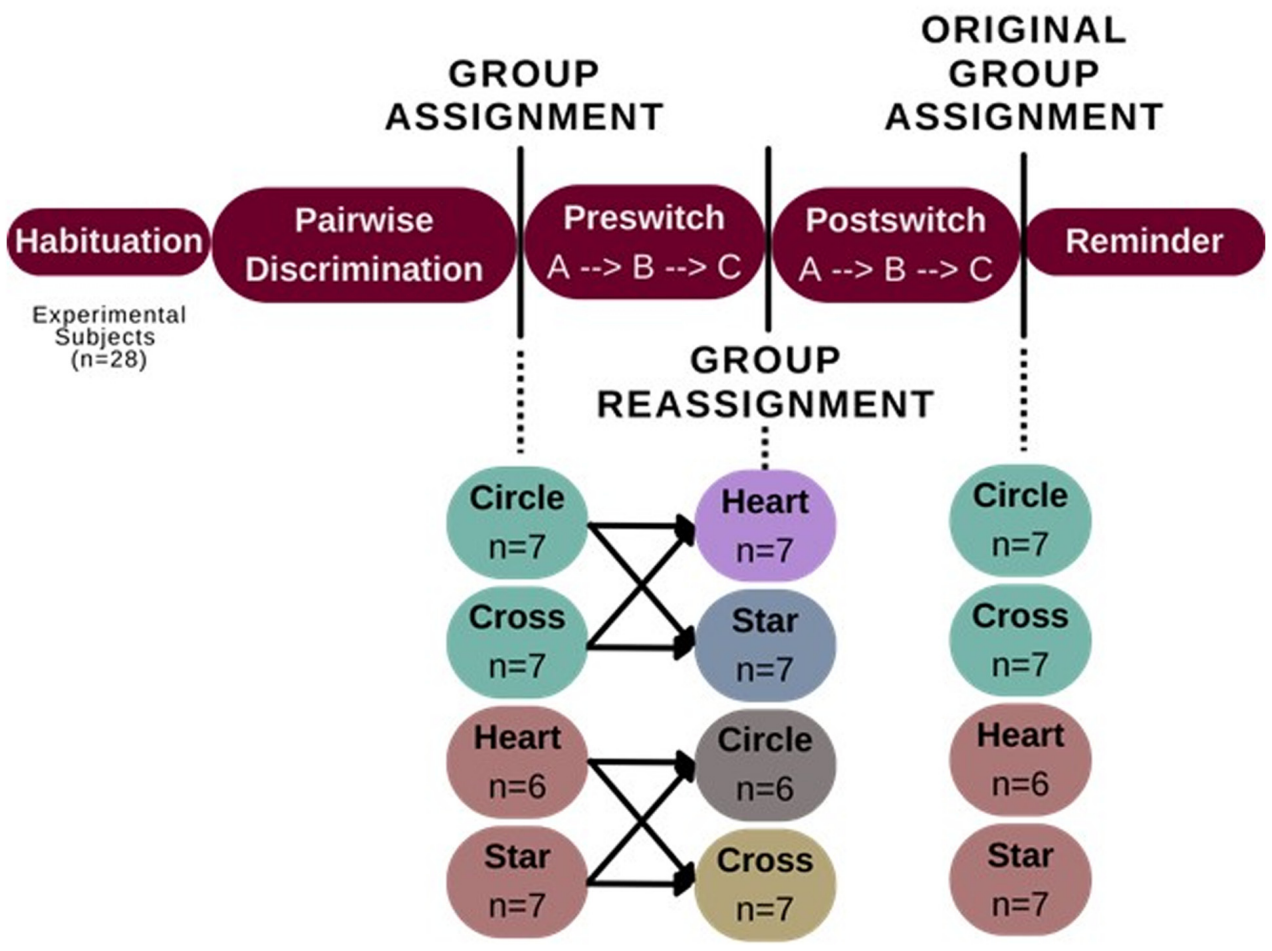
A breakdown of group sizes as described by their correct image. The total number of subjects started at *n* = 28 during habituation to operant boxes. One mouse was removed from the study at the end of the pairwise discrimination paradigm for failing to learn the task, so *n* = 27 started at the preswitch stage, as described above. Mice were then pseudorandomized into correct image groups (group 1 = circle/cross; group 2 = heart/star), with graphic examples provided in Figure 5. All 27 mice finished testing successfully at a criterion of at least 70% correct.

To begin preswitch stage A of the rule-learning task, all animals were assigned to one of two image set groups: circle/cross (*n* = 14) or heart/star (*n* = 13). These subject assignments were pseudorandomized but counterbalanced using pretraining pairwise discrimination scores to ensure that groups were equivalent. Subjects were further assigned (counterbalanced) within groups to either the circle/cross or the heart/star as target. Later, when moving into the postswitch stage, subjects were again divided evenly and counterbalanced into new target stimulus groups such that an equal number of animals with the circle as their correct image moved into both the heart and star, etc. (see Fig. 4). During testing, we found that animals learned to discriminate the heart/star image set faster than the circle/cross. Thus, stage A (described below) for the circle/cross set took slightly longer (both preswitch and postswitch) than the heart/star stage A. However, since days of testing were equalized across preswitch and postswitch within each set and since all subjects were tested on both sets, these apparent differences in difficulty were balanced out.

#### Preswitch—stage A (P79)

**Figure 5.**
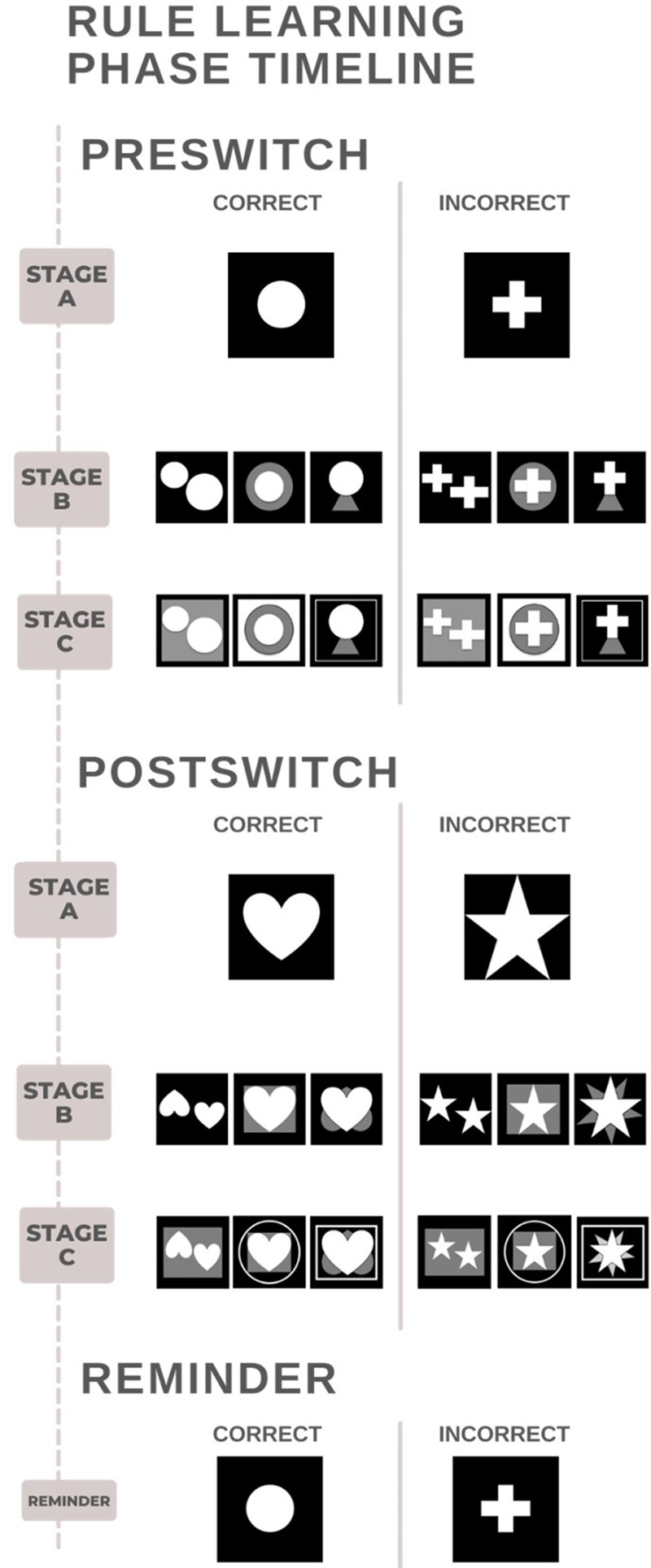
A sample time line of a mouse that started with the image set of circle/cross and a correct image of circle. Order and presentation of the first image and the correct image were counterbalanced across subjects. During each trial, one image was presented and all three images in stages B and C (preswitch and postswitch) were presented an equal amount.

Each subject began preswitch stage A (novel pairwise testing) with one session per day (a session equaling 60 min or a maximum of 30 trials completed). Subjects were again required to identify (touch) their target among two white images on a black screen. Possible targets were a circle, cross, heart, or star (see Fig. 5), and the side of the target presentation for each trial was randomized (Abbett II Software). Correct responses were rewarded with a 20-µL delivery of liquid reward, while incorrect responses were punished with a flash of the bright light (60 lumens) above the operant area and a time-out of 5 sec. Session results included the number of correct responses, the number of incorrect responses, and the total duration of each session. Once all subjects in a cohort (including both target groups: circle and heart or cross and star) met a criterion of 75% correct responses for at least 2 d, the entire cohort was moved to the next stage of testing.

#### Preswitch—stage B (P96)

In this stage, a new “distractor” image was introduced to both screen images during a trial. Mice were required to extract their target (the same as in stage A) from the distractor and identify it via screen touch (as before) to complete the session (see Fig. 5). Again, once both groups within a cohort reached a criterion of 75% correct responses over at least 2 d, the entire cohort was moved to the next stage of the task.

#### Preswitch—stage C (P106)

In this stage, an additional distractor was added to both screen images on each trial (see Fig. 4), while subjects continued to be required to extract and identify their target. Again, once both groups in a cohort reached a criterion of 75% correct responses over at least 2 d, mice in the cohort were allowed to move to the next stage of the task.

#### Postswitch—stages A–C (P116)

In postswitch testing, the set image pairs were swapped across groups (i.e., animals previously discriminating circle/cross were now assigned to heart/star and vice versa). Cohorts were run through the same stages of testing (A–C) for the new image sets using the same number of days per stage as used in preswitch testing for the other group. This was important both to balance exposure time within image set across cohorts and to allow statistical comparison of pre/postperformance for each image set.

#### Reminder (P143)

This stage of testing required subjects to go back and identify their original preswitch stage A target image. Subjects were presented with image pairs from preswitch stage A testing (circle or cross, or heart or star), with correct identification of their original target leading to a reward. As in all prior stages, image presentation was counterbalanced for side, and incorrect responses triggered a time-out (as above). Notably, this testing occurred ∼80 d after subjects were first placed in preswitch stage A.
